# Controlled compensation via non-equilibrium electrons in ZnO

**DOI:** 10.1038/s41598-018-35178-w

**Published:** 2018-11-19

**Authors:** Xiuhua Xie, Binghui Li, Zhenzhong Zhang, Shuangpeng Wang, Dezhen Shen

**Affiliations:** 1 State Key Laboratory of Luminescence and Applications, Changchun Institute of Optics, Fine Mechanics and Physics, Chinese Academy of Sciences, Changchun, 130033 People’s Republic of China; 2 Institute of Applied Physics and Materials Engineering, University of Macau, Macao, SAR 999078 China

## Abstract

Doping wide-band-gap semiconductor with impurities always accompanied spontaneous compensation of opposite charged intrinsic defects, which lead to invalid control of the type of free carriers. We demonstrate an effectual route to overcoming such detrimental defects formation during doping by suppressing Fermi level shifting using non-equilibrium carriers gathering on the polar epitaxial surfaces. Non-equilibrium carriers are generated by ultraviolet light excited interband transitions (photon energy greater than bandgap). Because the p-type dopants are compensated by non-equilibrium electrons at metal-polar surfaces, donor-type native defects are inhibited. This new doping strategy provides an attractive solution to self-compensation problems in wide–band-gap semiconductors with spontaneous polarization of the future.

## Introduction

Imperfections in semiconductors, such as defects and impurities, dictate their conductivity properties. The success of doping technology (imperfections levels controlling) has promoted semiconductor-based technologies, including light-emitting diodes (LEDs), microelectronics, photovoltaics, or, more recently, spintronics^1–4^. However, not all materials can be effectively doped to achieve both p-type and n-type, for example, halide perovskites and some wide-band-gap semiconductors^5,6^. One of the most thorny issues was the p-type doping of zinc oxide (ZnO)^7–9^, a naturally n-type material under normal growth conditions^7,10^. As the most promising candidate for ultraviolet laser materials, ZnO has been researched for decades in p-type doping^8,9,11,12^. It was once thought that sufficiently reducing native defects and unintentional incorporated impurities could induce ZnO to be doped effectively and thus achieving p-type conductivity-promoting^13,14^. However, there is one more main obstacle: Le Chatelier-type compensation (self-compensation effect) creates native donor-type defects (hole killers) that precisely negate the acceptor-type dopant introduced by external doping, while the Fermi level is pinned on the position near the middle of bandgap^6,9,15^. It is well known that for a donor-type native defect *D* at charge state *q* in a semiconductor, the defect concentration can be obtained by:

1 $$n(D,\,q)={N}_{0}{e}^{-\frac{{\rm{\Delta }}{H}_{f}(D,q)}{{k}_{B}T}}$$

where *N*_0_ is the total concentration of potential sites that can form *D* under charge state *q*, *T* is the absolute temperature, *k*_*B*_ is Boltzmann constant, and ${\rm{\Delta }}{H}_{f}(D,\,q)$ is the defect formation energy, which can be expressed officially as:

2 $${\rm{\Delta }}{H}_{f}^{(D,q)}({E}_{F},\mu )={\rm{\Delta }}{E}^{(D,q)}({E}_{F}=0,{\mu }_{i}=0)+\sum _{i}{n}_{i}{\mu }_{i}+q{E}_{F}$$

where ${\rm{\Delta }}{E}^{(D,q)}({E}_{F}=0,{\mu }_{i}=0)$ is the formation energy when the Fermi level (*E*_F_) and chemical potentials (*μ*_*i*_) are both equal to 0, which mean *E*_F_ is at the valence band maximum while *μ*_*i*_ of the atom *i* has the energy of the atom in the bulk form.

According to formulas 1 and 2, the concentration of native donor-type defects was governed by dual factors, *E*_F_ determined by free carriers and *μ*_*i*_ controlled by the external growth condition. Therefore, in principle, by adjusting *E*_F_ and *μ*_*i*_, the formation of donor-type native defects can be suppressed, thereby reducing the self-compensation effect in the p-type doping process. Unfortunately, in reality, in order to ensure the quality of crystals during the epitaxial growth of materials, the optional range of chemical potentials is very limited^16,17^. At the same time, the Fermi level cannot be arbitrarily changed, namely that in order to reduce the formation of donor-type native defects, the *E*_F_ should be rising, which contradicts the actual p-type doping^10^. Furthermore, *E*_F_ and *μ*_*i*_ are usually interdependent which means they cannot be independently varied to adjust defects formation energy.

Now that the hole-producing acceptor dopant in a semiconductor makes *E*_F_ approaches to the valence band edges, reducing the formation energy of donor-type native defects, an effective and feasible way to inhibit self-compensation is to overcome the *E*_F_ shift while incorporating the p-type dopant, which is very difficult to achieve. A successful example of this is the passivation of Mg-doped GaN with hydrogen (H)^1,18^. Due to the formation of Mg-H, the Fermi level of Mg-doped GaN cannot move toward the valence band maximum, thereby suppressing the spontaneous formation of nitrogen vacancies (compensating defects). However, H must be annealed out, subsequently, to realize the p-type conductivity^1,5^. Since H is essentially an electron donor, an alternative approach, limiting the shift of *E*_F_, is to directly provide electrons for compensating dopants during the p-type doping. In general, non-equilibrium electrons can be generated by thermal excitation. However, due to the wide bandgap (*E*_g_) of ZnO (3.37 eV), the concentration of electrons generated by thermal excitation, governed by mass action law (${n}_{i}=\sqrt{{N}_{C}{N}_{V}}{e}^{-\frac{{E}_{g}}{2{k}_{B}T}}$), is very low (about 10^12^ cm^−3^ at usual growth temperature 650 °C, far below the doping range 10^18^ to 10^20^ cm^−3^). Moreover, non-equilibrium holes are equally generated in the same region of the semiconductor. Taking into account the above two aspects, the thermal excitation process cannot suppress the shift of *E*_F_ induced by p-type doping. Therefore, an alternate excitation strategy should be sought, while efforts should be made to separate the generated electron from the hole.

**Figure 1:**
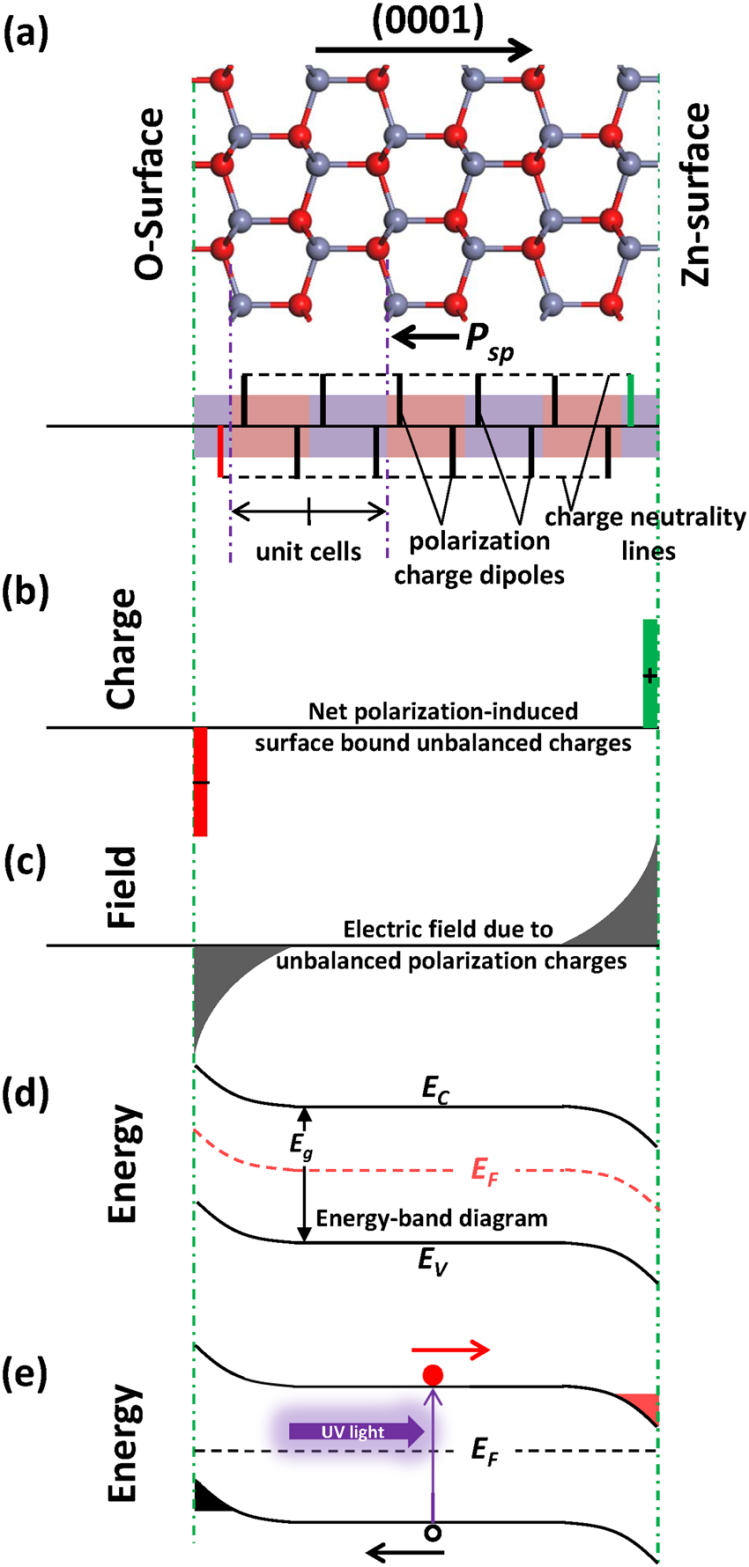
Schematic of suppression self-compensation in ZnO polar surfaces via non-equilibrium carriers excited by UV light. (**a**) Charge dipoles in every unit cell of the ZnO. The surface bound unbalanced charges induced by dipole breaks at the surfaces are shown in (**b**), which is the origin of the electric field in (**c**), and causing the energy-band bending in (**d**). Non-equilibrium carriers excited by UV light in (**e**) are driven into the both side polar surfaces, respectively, which leads to Fermi level shifting at the surfaces.

The non-centrosymmetric wurtzite crystal structure of ZnO, combined with the large ionic component of the Zn-O bonds, induces giant spontaneous polarization fields in ZnO^19^. As illustrated in Fig. 1(a), the spontaneous polarization can be depicted as charge dipoles in every unit cell of ZnO. At both of ideal termination surfaces (Zn-surface and O-surface), the abrupt discontinuity in the spontaneous polarization field causes the production of net unbalanced bound charges σ on both surfaces, Fig. 1(b), which determined by the Gauss law boundary conditions $\sigma =\overrightarrow{n}\cdot (\overrightarrow{{p}_{1}}-\overrightarrow{{p}_{2}})$, where ($\overrightarrow{{p}_{1}}$, $\overrightarrow{{p}_{2}}$) are the polarizations across the surfaces and the $\overrightarrow{n}$ is the unit vector perpendicular to the surfaces. Due to Zn-surface forming a positive bound charge, a high positive electric field is generated at this region and the energy band is bent downward, and vice versa, as shown in Fig. 1(c,d). Under the premise of high crystalline quality of ZnO^14^, there are no sufficient residual carriers to screen this surface polarization electric field. In the case of ultraviolet (UV) light excitation (with photon energy greater than *E*_g_), as illustrated schematically in Fig. 1(e), non-equilibrium electrons generated by interband transitions will drive to the Zn-surface, which leads to *E*_F_ increasing. When p-doped ZnO is grown along the [0001] orientation (Zn-face) under UV light, the epitaxial surface is always under electron-rich conditions, ie the downward trend of *E*_F_ is suppressed. Therefore, the formation of donor-type defects will be inhibited even under p-dopant incorporation. Unlike p-type doping of GaN which requires H-removal by annealing following Mg-H complex formation, the removal of non-equilibrium carriers in ZnO is accomplished simply by turning off the UV illumination. This work demonstrates the ability to suppress the self-compensation via non-equilibrium electrons excited by UV light.

In this study, intrinsic and nitrogen (N) doped ZnO films were grown on 5 μm-thick (0001) GaN/c-sapphire templates. The substrates were thermally cleaned at 650 °C for 12 hours prior to loading into the molecular beam epitaxy (MBE) growth chamber. To ensure uniform Zn-polar oriented growth, the surfaces of (0001) GaN templates were pre-exposure to Zn atom beam to prevent the oxidation when growth starting^20,21^. Conversely, for O-polar oriented growth, the gallium oxide (Ga_2_O_3_), obtained by O pre-exposure, between GaN and ZnO was essential. Inversion symmetry of Ga_2_O_3_ leads to invert polarity from the Ga-polar to O-polar^20^. UV light, generated by the custom-built UV LEDs array (peak wavelength 365 nm) with total optical power 600 W, was irradiated to the substrate through one of the MBE cell port with a sapphire window. Considering that the area of the LEDs array is 16π cm^2^, the light directivity is 60°, and the distance from the LEDs array to the substrate is 60 cm, the photon (energy above *E*_g_) density of the irradiated ZnO surface is about 1.7 × 10^17^ cm^−2^ in the epitaxial process. Even considering absorption efficiency, recombination, etc., the surface density of photogenerated electrons is greater than the surface density of dopant atoms (10^12^ to 10^13^ cm^−2^ corresponding doping concentration 10^19^ to 10^20^ cm^−3^), which are sufficient to compensate the acceptors.

**Figure 2:**
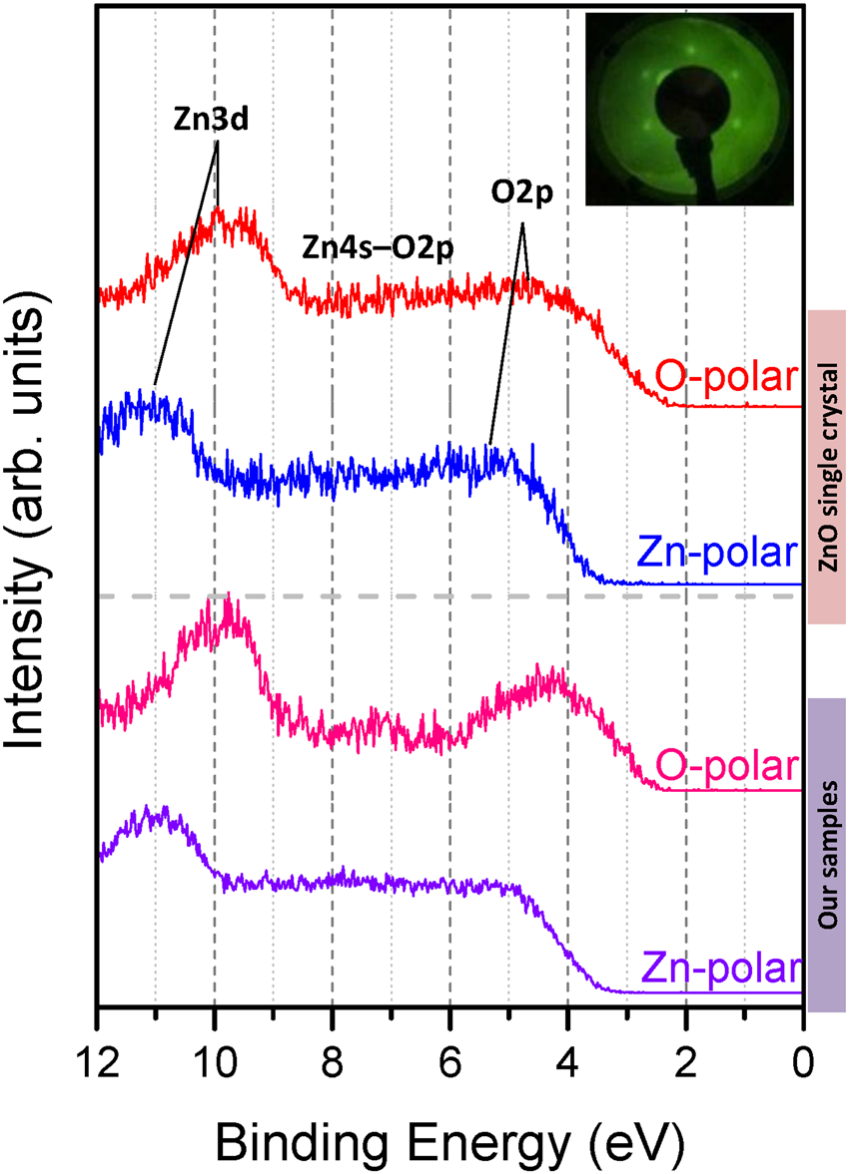
UPS spectra of ZnO films. The valence band maximum are shifted further away from the Fermi level for the Zn surfaces compared to the O surfaces, whether for single crystals or our samples. The inset on the top right corner shows the pattern of LEED.

To comprehend how the energy band bending of the polar surfaces was affected by surface net unbalanced bound charges, ultraviolet photoelectron spectroscopy (UPS) was employed to determine positions of the valence band edge. Due to the short electron mean free path when using the He I-line (for ZnO about 1 nm), the valence band spectra mainly indicates the topmost surface layer properties. For comparison purpose, there was also a group of ZnO single crystal (Zn-polar and O-polar, Tokyo Denpa Co. Ltd.) measured. All samples were cleaned by cycles of 3 keV Ar^+^ ion sputtering and annealing to 700 °C in ultra-high vacuum (UHV) prior to analysis. As can be seen from the low-energy electron diffraction (LEED), inset of Fig. 2, the surfaces of all cleaned sample exhibit a typical (1 × 1) surface, which indicates that the band bending cannot result by surface reconstruction or contamination. The known feature states belonging to the O 2p, Zn 4s-O 2p and Zn 3d band can be easily identified from the valence band spectra (illustrated in Fig. 2). The valence band maximum of Zn-polar surfaces is shifted away from *E*_F_ compared to the O-polar surfaces. Such shifts occur owing to the electrostatic field induced by the net unbalanced bound charges at the polar surfaces.

**Figure 3:**
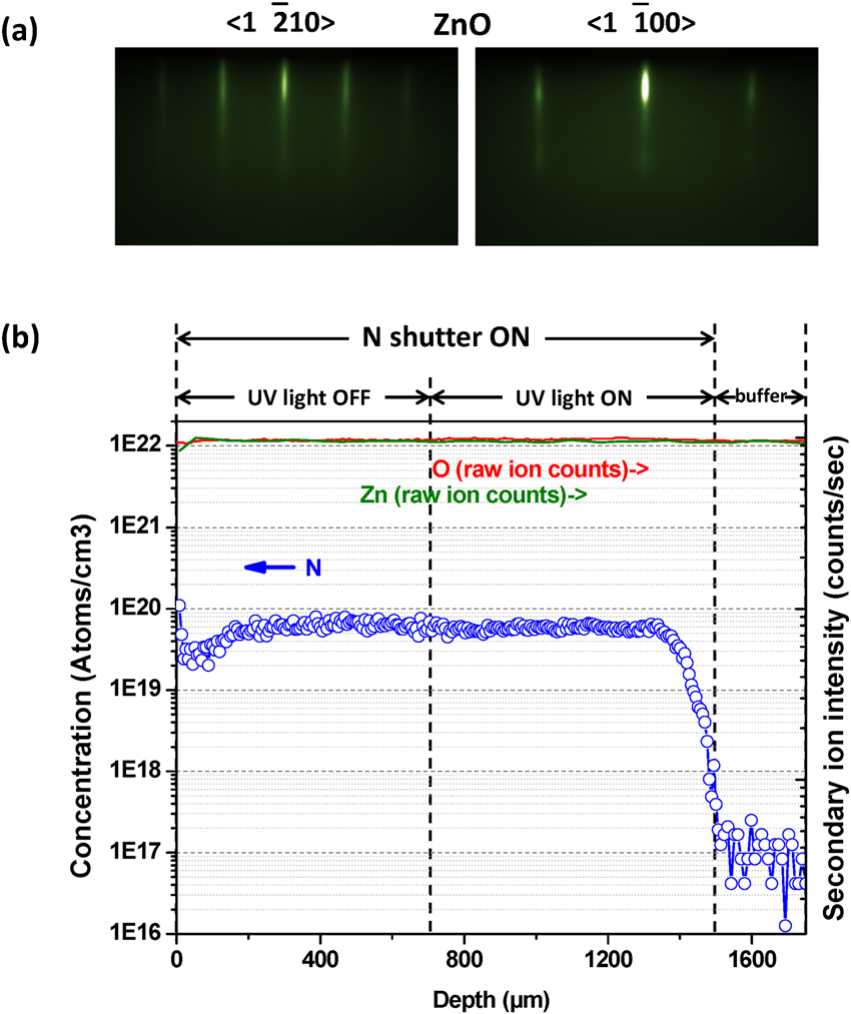
RHEED patterns along <1$\bar{2}$10> and <1$\bar{1}$00> electron beam azimuths show the (**a**) desirable (1 × 1) ZnO surface. Sharp streaky RHEED patterns indicate the achievement of a two-dimensional ZnO growth front surface morphology, during the epitaxy. Secondary ion mass spectroscopy (SIMS) depth profile for ZnO doped with N is shown in (**b**). N doping concentration is consistent in both UV on/off layers.

Figure 3(a) shows the real time growth front condition of Zn-polar surfaces monitored by reflection high-energy electron diffraction (RHEED). Because of the small glancing angle (<3 degrees) of accelerated electron beam (20 keV), the diffraction pattern of reflected electrons indicates the surface structure and growth dynamics of top monolayers. The growth front of Zn-face exhibits a two-dimensional growth model under the desirable (1 × 1) surface. There were no surface reconstructions take place during the whole epitaxy growth process. As can be seen in Fig. 3(b), N doping concentration is about 7 × 10^19^ cm^−3^ which is consistent throughout the doped layer. After growing the ZnO buffer layer, the doping process is equally divided into two stages. That is, the initial UV light-on stage and the normal doping stage after it. Based on previously analyzed, the spatial distribution of oxygen vacancies (*V*_O_), major compensating defects in ZnO, will be affected by ultraviolet light (see further discussion below).

**Figure 4:**
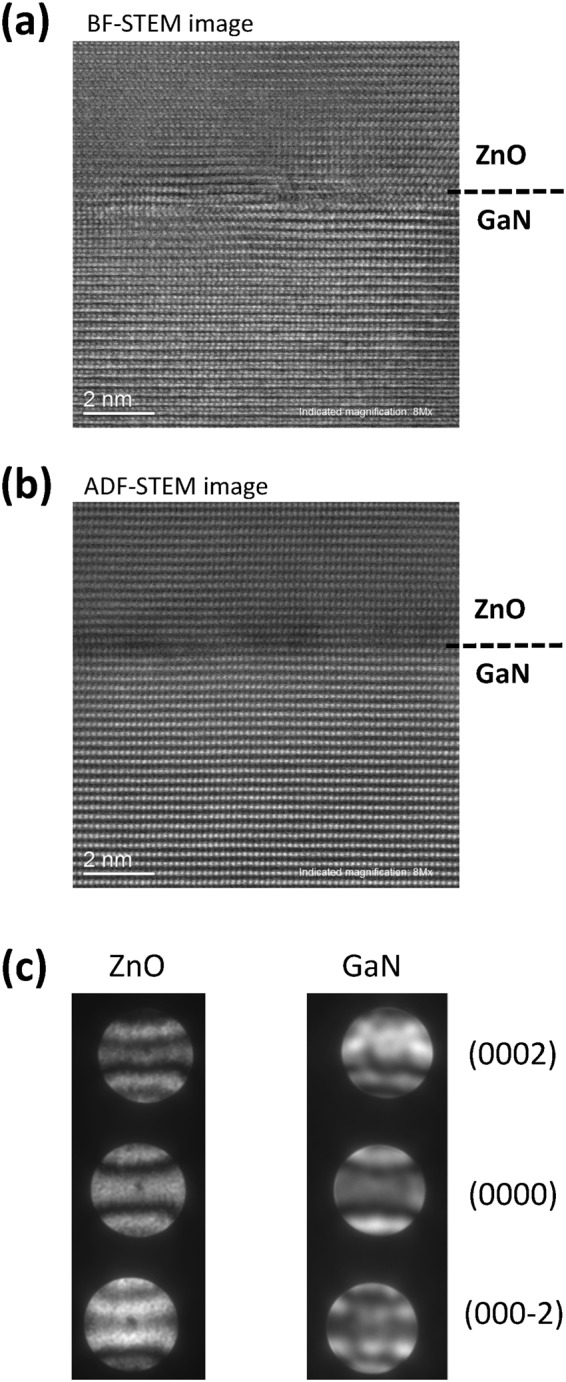
(**a**) Cross-section BF and ADF STEM images of the interface between ZnO and GaN. (**b**) CBED patterns of ZnO and GaN.

Figure 4(a,b) show bright-field (BF) and annular dark-field (ADF) scanning transmission electron microscopy (STEM) images of the interface between ZnO and GaN. A well-defined interface without the oxide layer was obtained by using Zn pre-exposure, which is the basis for ensuring Zn-face polarity. The epitaxial ZnO layers are high crystalline quality. The polarity of ZnO and GaN further determined by convergent beam electron diffraction (CBED). As illustrated in Fig. 4(c), the ZnO layers have the [0001] growth orientation, which is same as the GaN substrate.

**Figure 5:**
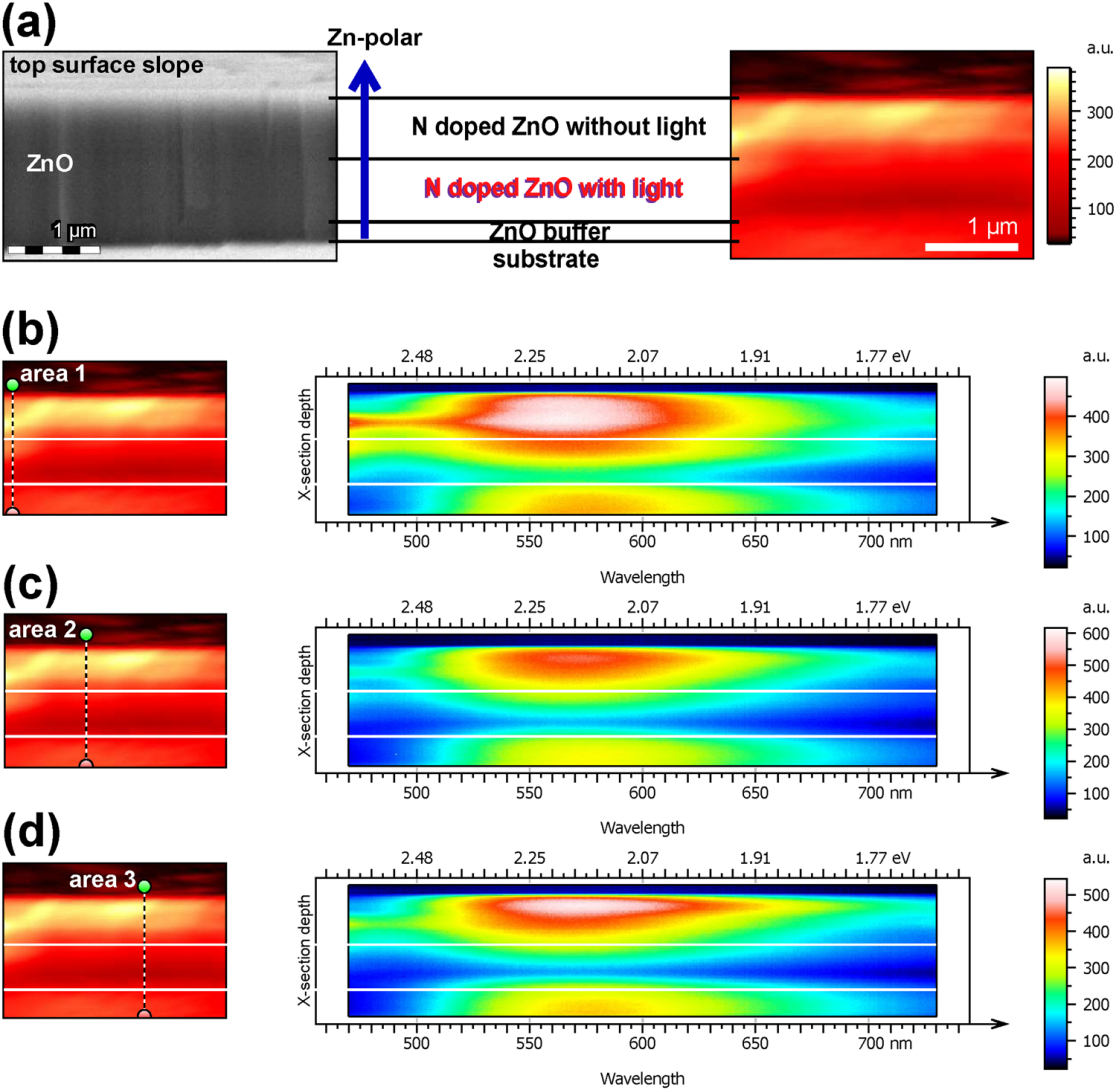
(**a**) SEM image of ZnO in cross section and hyperspectral CL map of the same cross section colour-coded by emission intensity. The spatial distribution of defects luminescence in ZnO layers is clear in the CL dataset. There are three areas (**b**–**d**) of the extracted spectral profiles (linear scale) across selected areas. Oxygen vacancies-related defect luminescence is mainly concentrated in layers that are not irradiated by UV light during epitaxy.

Figure 5 shows the 450–730 nm range cathodoluminescence (CL) cross section mapping of ZnO layers under the conditions of electron beam energy 10 keV and at room temperature. A very obvious spatial inhomogeneous distribution of defects luminescence intensity can be observed. As can be seen in Fig. 5(a), defects luminescence intensity of N-doped ZnO layer without UV light is stronger than the doped layer with UV light. According to previous studies, the luminescence range of 500–650 nm was caused by *V*_O_^22–24^. As illustrated in Fig. (b–d), the three areas of the extracted spectral profiles all exhibit that *V*_O_ concentrate on the top N-doped layer in which the UV light is keeping off during the doping process. A weak *V*_O_ signal is also shown in the ZnO buffer layer, which may be caused by poor crystallization quality at the initial heteroepitaxy. The layer of N-doped ZnO with UV light shows very weak CL signal of *V*_O_. Due to an excitation through carriers diffused into the top layer and the buffer layer, the boundary between each layers was not sharp. Considering that the N concentration is constant and other growth conditions are stable, we believe that the main cause of the non-uniform spatial distribution of *V*_O_ is the switching of UV light. That is, due to the action of the surface polarization electric field, the non-equilibrium electrons are allowed to accumulate on the Zn-polar surface, and thus compensation for N is achieved, thereby suppressing the generation of *V*_O_.

In conclusion, we demonstrate controlled compensation of p-type doping ZnO by exciting non-equilibrium electrons using UV light with photon energy above *E*_g_. The formation of *V*_O_ is suppressed due to the non-equilibrium electrons acting on the Zn-polar epitaxial surfaces. Non-equilibrium carriers in polar surfaces provide an attractive solution to self-compensation problems in wide–band-gap semiconductors with spontaneous polarization of the future.

## Methods

### Films Growth

Intrinsic and N doped ZnO films were grown by MBE system (DCA-P600, Finland), which equipped with the oxford radio-frequency (RF) atom source (13.56 MHz, HD25) with ion removal control for active O and N atom, and the Knudsen effusion cells for Zn. The RF of O and N were operating at 320 W and 350 W, respectively. O_2_ flow rate was varied in the ranges of 1.50–2.32 sccm while N_2_ 0.76 sccm. The beam equivalent pressure of Zn was 1 × 10^−7^ Torr which measured by using a nude ionization gauge working in front of the substrate. The growth temperature is 550 °C, measured by the thermocouple on the back of the substrate. UV light was generated by the custom-built UV LEDs array (peak wavelength 365 nm) with total optical power 600 W, which works under water cooling conditions to ensure a stable optical power output.

### Characterization

The energy band bending of the intrinsic ZnO polar surfaces was characterized by ultraviolet photoelectron spectroscopy (UPS, PREVAC, under He I-line 21.2 eV irradiation). During epitaxy, the surface of ZnO was monitored *in situ* by RHEED. N depth profile was measured by using secondary ion mass spectroscopy (SIMS, CAMECA IMS-7F, primary ion beam cesium 133), which has been calibrated with a standard sample. The lattice arrangement and polarity orientation of ZnO films were characterized by scanning transmission electron microscopy (STEM, Hitachi HF3300). The depth profile of compensating defects, *V*_O_, was measured by utilizing high spatial resolution cathodoluminescence (CL, Attolight Rosa 4634 CL microscope). The CL microscope system has the objective lens of a field-emission-gun scanning electron microscope (FEG-SEM), within tightly integrates a high numerical aperture (NA = 0.72) achromatic reflective lens. The focal plane of the optical lens matches the optimum working distance of FEG-SEM.
